# The Therapeutic Effect and In Vivo Assessment of Palmitoyl- GDPH on the Wound Healing Process

**DOI:** 10.3390/pharmaceutics13020193

**Published:** 2021-02-01

**Authors:** Nur Izzah Md Fadilah, Mohd Basyaruddin Abdul Rahman, Loqman Mohamad Yusof, Noordin Mohamed Mustapha, Haslina Ahmad

**Affiliations:** 1 Integrated Chemical Biophysics Research, Universiti Putra Malaysia, Serdang 43400, Selangor, Malaysia; nurizzahfadilah@gmail.com (N.I.M.F.); basya@upm.edu.my (M.B.A.R.); 2 Department of Chemistry, Faculty of Science, Universiti Putra Malaysia, Serdang 43400, Selangor, Malaysia; 3 Department of Companion Animal Medicine and Surgery, Faculty of Veterinary Medicine, Universiti Putra Malaysia, Serdang 43400, Selangor, Malaysia; loqman@upm.edu.my (L.M.Y.); noodinmm@upm.edu.my (N.M.M.)

**Keywords:** peptide, fatty acid, therapeutic agent, wound healing, topical drug

## Abstract

The standard treatment of open wounds via the direct usage of therapeutic agents is not without limitations with respect to healing. Small peptides can create a favorable milieu for accelerating the healing of wounds. This study presents the potential of a novel fatty acid conjugated tetrapeptide (palmitic acid-glycine-aspartic acid-proline-histidine; Palmitoyl-GDPH) in alleviating wound healing. Tetracycline was employed as a standard control drug following its significance in wound healing including biologically active and antimicrobial effects. The peptide in liquid form was applied on to a 4 cm^2^ full thickness wound surgically induced at the dorsum of Sprague Dawley (SD) rats. The in vivo wound treatment with Palmitoyl-GDPH for eighteen days, histologically demonstrated an almost perfect healing exhibited by increased re-epithelialization, enhanced collagen deposition, and diminished scar formation compared to the controls. In addition, the well-developed epidermal-dermal junction and ultimate stimulation of hair follicle-growth in the Palmitoyl-GDPH treated group indicated the wound to have healed as functionally viable tissues. In general, the much lower hemogram values in the Palmitoyl-GDPH group indicated that the ongoing healing is en route to an earlier recovery. Additionally, the liver, kidney, and pancreas function biomarkers being within normal limits indicated the relatively non-toxic nature of Palmitoyl-GDPH at the used dosage. These results indisputably supported the great potential of this newly synthesized Palmitoyl-GDPH to be used as an effective therapeutic agent for wound healing (this actually means creating a new wound).

## 1. Introduction

Skin is the largest organ that covers the body and acts as a physical barrier between the inner and outer environment. As an external barrier, it is always exposed to the adverse effects following physical injury such as infection, shock, and even death [1]. Therefore, it is vital to the body to mount an immediate response to the injury in order to restore the skin function through an effective wound healing process. Wound healing is a complex and interactive process that involves four stages, which are hemostasis, inflammation, proliferation, and remodeling [2]. Wound healing starts at the time injury occurs and progresses for different lengths of time based on the degree of the injury [3]. Current therapeutic drugs and delivery systems have been extensively investigated for wound healing [4,5,6]. However, there are some limitations of the current therapies including healing time, itching, irritation, dryness, scar formation, and high treatment cost [7]. Therefore, it is necessary to develop more efficacious and active agent to improve and expedite wound healing leading to a reduction of the total treatment costs.

The use of peptides as therapeutics has been formulated over time in drug development. The study of bioactive peptides with therapeutic efficacy has demonstrated some beneficial uses in medicinal chemistry field and aroused the interest of many researchers [8]. Recently, the therapeutic activity of antimicrobial peptides (AMPs) is noteworthy for tissue and wound healing as they are used in the treatment of microbial infections. They are produced by epithelial surfaces and have been described as mediators of the body’s innate defense response that directly kill or inhibit the growth of microorganisms. The AMPs are the short stretch of amino acids that have broad-spectrum antimicrobial activity. In the case of skin injury, AMPs expression is highly up-regulated at the wound edge due to increased synthesis by keratinocytes and deposition from degranulation of recruited neutrophils, indicating that AMPs are a potential wound-healing stimulator [9,10,11]. Nevertheless, the insect AMP cecropin-derived peptide HB-107 lacks antimicrobial activity but when applied to mouse wounds, it induces keratinocyte hyperplasia and increases leukocyte infiltration. Furthermore, HB-107 stimulates interleukin-8 secretion from cultured endothelial cells, an effect that may explain the increase in leukocyte migration. These findings demonstrated that non-antimicrobial peptides could function as effectors of cutaneous wound repair [12]. Consequently, the demand for bioactive peptides is increasing for wound treatment applications, particularly with the emergence of advanced technologies such as modification through amino acids, and incorporation and conjugation of moieties. The desired characteristics of a novel therapeutic for the infected wound treatment would include a broad-spectrum antimicrobial activity, low cytotoxicity, and strong wound closure activity that can withstand the host environment.

At the forefront of wound healing therapeutics, bioactive peptides are often considered for the therapeutic development of wound healing agents. In particular, peptides perform important functions in the skin such as inhibit collagen and stimulate fibroblast production. Peptides are used in-situ and topically applied as a drug for wound healing due to their availability, solubility, and effectiveness. Therefore, lower dosage is needed for more efficient topical delivery. By comparison, a protein is a large molecule which contains more than 50 amino acids (high molecular weight), while a peptide is a small, short molecule consisting of between 2 to 50 amino acids and having low molecular weight [13]. In a previous study, a designed small peptide (tiger17, c[WCKPKPKPRCH-NH_2_]) containing only 11 amino acid residues was proven to be a potent wound healer. It showed strong wound healing promoting activity in a murine model with a full thickness dermal wound. The peptide tiger17 exerted significant effects on three stages of wound healing progresses in a murine full-thickness skin wound model, including the recruitment of macro-phages to the wound site during the inflammatory reaction stage, a promotion of the migration and proliferation of both keratinocytes and fibroblasts leading to re-epithelialization, and granulation tissue formation and tissue remodeling [14]. Besides, another study reported that a bioactive peptide of 19 amino acid residues named SHAP1 (APKAMKLLKKLLKLQKKGI) showed stronger wound closure activity. In vivo analysis revealed that SHAP1 treatment accelerated closure and healing of full-thickness excisional wounds in mice. Moreover, SHAP1 effectively eliminated *Staphylococcus aureus* infection and enhanced wound healing in murine *Staphylococcus aureus*-infected wounds [15].

Fatty acid conjugated peptide, along with its conjugation, was a compound from the reaction of amino acid residues with a fatty acid. Conjugation is a new and popular concept in order to minimize long sequence and enhance the properties of peptide drug candidates [16]. This type of therapeutic agent extensively demonstrates efficacy in wound healing since the fatty acid as a chemical penetration enhancer which improve topical delivery across the epidermis [17,18]. There are some fatty acid conjugated drugs have been approved by U.S. Food and Drug Administration (FDA) for clinical applications such as liraglutide and insulin detemir (myristic acid conjugated to lysine side-chain) [19]. RIGIN^TM^ is a market product developed by SEDERMA which is also known as Palmitoyl tetrapeptide-7, with the sequence of Gly-Glu-Pro-Arg (Palmitoyl-GQPR). It is used in skin care products and works as an anti-inflammatory agent and has anti-aging and skin firming abilities [20]. Besides, lipoic acid conjugate Lys-Thr-Thr-Lys-Ser (KTTKS) has effective skin whitening and anti-aging agents [21]. Moreover, other studies described positive effects of fatty acids in wound healing [22,23,24,25]. They reported that palmitoyl fatty acid can alter skin structural and immunological status since they constitute the stratum corneum and thus, improve the permeability of the skin. This is correlated to the acceleration of wound closure since it shortens the bleeding time and responds to stabilize the fibrin as a consequent of fibroblast migration [26].

Up to date, the therapeutic small peptide with less than five amino acid residues for wound treatment is still in its infancy stage and has not yet been fully exploited in clinically. Of particular interest is the short sequence of peptide which is palmitic acid conjugated glycine- aspartic acid- proline- histidine, referred to as Palmitoyl-GDPH, which possesses strong proliferative activity and promoted migration towards normal human dermal fibroblast (NHDF) cells [27]. With this in mind, we hypothesized that Palmitoyl-GDPH would be a novel approach in the treatment of wounds. In the present study, we explore the in vivo therapeutic effects of Palmitoyl-GDPH on full-thickness excision wound model, including histological and hematological studies. The antibiotic tetracycline that is commonly used to prevent bacteria and consequently promote faster healing rate was selected to compare the wound healing efficacy of the Palmitoyl-GDPH. It was one of the largest groups extensively used in human and veterinary medicine [28]. Based on the review of the literature, tetracycline enhances the rate of wound healing and has an antibacterial property, and has also been evaluated on the surgical wound healing process of animals especially for diabetic diseases. It was possible to observe tetracycline present beneficial effects for healing superficial infections because it had effects on the bacterial agent directly at the application site [29]. Since tetracycline is commercially available and commonly applied as antibacterial in wound treatment, it was used to compare the same effect of Palmitoyl-GDPH in wound healing as the tetracycline. Furthermore, histological, hematological, and selected biomarkers of the liver, kidney, and pancreas were also evaluated to assess the influence of Palmitoyl-GDPH treatment on healing process in experimental rats. The results obtained from this study will provide strong evidence that Palmitoyl-GDPH is a promising agent for wound treatment due to its potency for healing cutaneous wounds of Sprague Dawley rats.

## 2. Materials and Methods

### 2.1. Synthesis and Characterization of Peptide

Palmitic acid conjugated to Gly-Asp-Pro-His (Palmitoyl-GDPH) was synthesized by the solid phase peptide synthesis (SPPS) method. In the chromatographic profile, it was observed to have reached a significantly high peak at retention time 10.73 min, corresponding to the peptide with a percentage purity of 98.59% as reported previously. The Palmitoyl-GDPH was freshly prepared with a concentration 12.5 µg/mL in liquid form. The concentration chosen for this in vivo study was based on the previous wound scratch assay conducted where we treated the cells with different concentrations from 12.5 µg/mL to 100 µg/mL. According to the data obtained, the cells achieved full gap closure within 48 h even treated with the minimum concentration [27]. The dry peptide is stable at room temperature for days but for long term storage, it is recommended to store it in lyophilized or powder form at −20 °C. However, it was important to allow the vial to warm up to room temperature before opening it as to avoid condensation of atmospheric water on the peptide. Also, sterilized gauze was used to cover the dorsum of the rat following the application of the peptide onto the wound. Palmitoyl-GDPH: Mass (Yield): 0.3587 g (77.8%). ^1^H nuclear magnetic resonance (NMR), dimethyl sulfoxide (DMSO, δ ppm): 8.94 (m, 1H), 8.25 (m, 1H), 8.11 (m, 1H), 7.98 (m, 1H), 7.42 (m, 1H), 4.84 (dd, 1H), 4.53 (m, 1H), 4.30 (dd, 1H), 3.68 (d, 6H), 3.17 (m, 1H), 3.04 (m, 1H), 2.64 (m, 1H), 2.42 (m, 1H), 2.10 (m, 2H), 1.98 (m, 1H), 1.83 (m, 2H), 1.49 (m, 2H), 1.25 (m, 26H), 0.86 (t, 3H).

### 2.2. Ethical Requirement

All the procedures adhered strictly to the approved guidelines of the Institutional Animal Care and Use Committee (IACUC) of the Universiti Putra Malaysia. The animal utilization protocol (AUP) was approved on 19 July 2018 and the certificate no. was UPM/IACUC/AUP-R032/2018.

### 2.3. Animals and Management

A total of eighteen healthy male Sprague Dawley (SD) rats with an average body weight of 250–300 g (7–8 weeks of age) were purchased from the Animal Resource Unit, Faculty of Veterinary Medicine, Universiti Putra Malaysia, Serdang, Malaysia. The rats were fed with a standard dry pellet diet and filtered drinking water ad libitum, placed individually in a cage in a standard room temperature of 22–23 °C and 12 h of artificial and natural light (alternating) with dark cycle at the Animal Research Facility (ARF), Faculty of Veterinary Medicine, Universiti Putra Malaysia, Serdang, Malaysia. All the experimental animals were acclimatized for seven days prior to the commencement of the study and they were labeled appropriately. Once the study commenced, clinical observation was made once a day for mortality, moribund, illness, or reaction to treatment such as changes in fur, dullness, restlessness, diarrhea, and coma [30].

**Figure 1:**
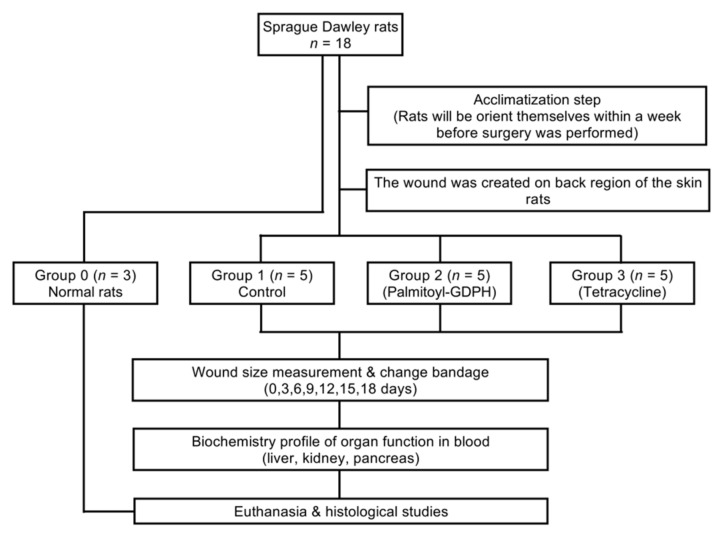
Flow diagram showing how the animals were used in this study.

The rats were randomly divided into four groups, viz; normal rats, Group 0 (intact skin, *n* = 3) while the remaining three groups comprised of five rats each of whose dorsum was surgically incised and applied with different of treatments as shown in Figure 1. Meanwhile, the dorsum of rats in Group 0 did not receive any treatment. Accordingly, those in Group 1, Group 2, and Group 3 were treated with 0.5% saline solution, Palmitoyl-GDPH, and standard drug-tetracycline, respectively. In order to minimize cross-contamination of treatments used and potential cross-infection, all rats were individually caged.

### 2.4. Excisional Wound

The health status of the rats was examined before surgery and only those deemed fit were used. The rats were anesthetized by intraperitoneal injection of 0.2 mL of Ketamine (80 mg/kg) and 0.1 mL of Xylazine (10 mg/kg) prior to the creation of wounds. The dorsal hair close to thoracic area of the rats was shaved using an electrical shaver followed by aseptic surgical preparation using Chlorhexidine, 70% ethanol, and tincture iodine solutions prior to the surgical skin excision. A full-thickness excisional wound of 2 cm × 2 cm was induced at the back region by using a scalpel blade [31]. The initial wound area was measured immediately by placing a tracing paper over the wound and then tracing the area onto graph paper. The developed wound was then treated with the designated treatment as stated earlier. Later, the wound was covered with a soft cotton gauze and adhesive tape. The dressing was replaced every three days (day 3, 6, 9, 12, 15, and 18 post-surgery) following wound area measurement. The wound was also treated topically every three days until day 18. The changes in wound size were observed, measured, and photographed. For post-operative analgesia, the rats were injected intramuscularly with 0.1 mL Meloxicam (1 mg/kg) once daily during the post-surgical days. At the end of the experimental period, all rats were euthanized with 0.2 mL of Pentobarbitone (150 mg/kg) via intraperitoneal route for histological tissue sampling and the carcass was disposed through the post-mortem facility [32].

### 2.5. Topical Application of Therapeutic Agent

The wounds of Group 1 rats were treated topically with 1.0 mL of normal saline solution every three days; this served as the control group. The wounds of Group 2 rats were treated topically with 1.0 mL of Palmitoyl-GDPH (12.5 µg/mL) every three days. Meanwhile, the wounds of Group 3 rats were treated topically with 1.0 mL of tetracycline (12.5 µg/mL) every three days and served as a reference standard control. The concentration used was the minimum concentration from the in vitro study as reported previously [27].

### 2.6. Determination of Body Weight Changes

The rats were observed daily throughout the experiment to ensure that their welfare was in good order in accordance with the ethical requirement. Likewise, the weight of rats was also taken every three days by using a sensitive weighing balance to observe any changes throughout the experiment [33].

### 2.7. Examination of Wound Contraction

Progressive changes in wound healing were assessed by tracing the wound area on transparent tracing paper on day 0, 3, 6, 9, 12, 15, and 18 post-surgery. Transparent paper was laid over the wound and we traced it using a permanent marker. Then, the wound area was quantified using graph paper by counting the number of squares covered within the wound area [3,34]. The percentage of wound contraction was measured according to the following Equation (1):

(1) $$\mathrm{Wound}\;\mathrm{closure}\;\mathrm{percentage}\;(\%)\;=\;(\frac{A_{0}-A_{t}}{A_{0}})\;\times \;100$$

where *A*_0_ was the original wound area and *A*_t_ was the wound area on day *t* post wounding.

### 2.8. Histological Analysis

#### 2.8.1. Tissue Preparation

After healing day 18 post wounding, skin samples measuring approximately 2 cm × 2 cm from the center of the wounded area and thickness of 5 µM were cut for all treatment groups at day 18 under anesthesia. For the normal group, the skin tissue was also cut with the same thickness of 5 µM. Histological slide preparations were based on the standard procedure [35]. The skin samples were immediately fixed in 10% neutral buffered formalin solution for 24 h. Then, the tissues were dehydrated with 70, 80, 95 and 100% alcohol and infiltrated by tissue processor machine. After 24 h, the tissues were embedded in solid paraffin block and trimmed until a complete and clear section was seen. A microtome was used for making 4 µM of tissue ribbon while water bath at temperature 60 °C was used in floating and fishing tissues onto the slides. The prepared slides were dried for 24 h until they were ready to stain.

#### 2.8.2. Hematoxylin and Eosin (H & E) Staining

A staining jar was used in this section in order to place the slides. The prepared slides were deparaffinized in absolute xylene for 5 min followed by 100% and 70% ethanol for 5 min of each percentage. Then, the slides were rinsed with running tap water for 2 min. Consequently, the slides were submerged into Harris’ Hematoxylin solution for 5 min and rinsed again with running tap water 2–3 times. The slides were dipped (3 dips) in 1% acid alcohol for 3 s to decolorize it and left under a running tap water for about 5 min. Next, the slides were submerged in Eosin for 1 min followed by spraying with 95% ethanol. After rinsing the slides under a running tap water (5–10 s), they were sprayed again with 95% ethanol to clean and then left to dry for 24 h at 38 °C. Finally, each slide was mounted with cover slip by using DPX (distyrene, plasticizer and xylene) mounting. The stained slides were ready to be observed under light microscope for the assessment of tissue formation and wound maturity [35].

#### 2.8.3. Masson’s Trichome Staining

Standard histological procedures were applied to all samples prior to the staining. In order to study the collagen deposition and their arrangement in the wounded tissue, the skin sections were subjected to the modified Masson Trichrome staining according to the method described by Suvik and Effendy [36]. Granulation skin tissue slides were placed in staining jar and deparaffinized by submerging them into absolute xylene for 5 min followed by 100% and 70% ethanol for 5 min each. The slides then were submerged in Bouin’s solution at 56 °C for 1 h. Next, the slides were cooled and washed under a running tap water until yellow color in the samples disappeared.

For the purpose of differentiating nuclei, the slides then immersed in Weigert’s iron hematoxylin solution for 10 min, and then washed under a running tap water for 10 min. The procedure was continued with immersing the slides into anionic dyes, acid fuchsin solution for 2 min in order to stain cytoplasm, then the slides were washed again under running tap water for 2 min. Next, the slides were treated with phosphomolybidic acid solution for 10 min and immediately submerged into methyl blue solution for 5 min in order to stain the fibroblast and collagen. After that, the slides were washed under running tap water for 2 min and later, they were treated with 1% acetic acid solution for 3 min followed by dehydrated into a series of ethanol of 70, 80, 95 and 100% for 1 min for each percentage. Lastly, each slide was mounted with cover slip by using DPX mounting and the stained slides were ready to be observed under light microscope.

### 2.9. Collagen Density Evaluation

The histological changes were viewed and captured under a light microscope MoticamPro 285A model (Motic, Version 1.1.0.335, Kowloon, Hong Kong, 2013) with the aid of a software image analyzer (Motic live imaging module). The measurements were made based on the intensity of the blue color which was directly correlated to the collagen density. It was measured under the wound area at 10× and 40× magnification and compared to skin of normal rats from Group 0. The mean of collagen values obtained for normal dermis was equivalent of 100 [35]. The collagen density of wound area was expressed in the ratio of percentage compared to collagen density of normal dermis during the post-wounding day as the following Equation (2):

(2) $$\mathrm{Ratio}\;(\%)\;=\;(\frac{\mathrm{Average}\;\mathrm{collagen}\;\mathrm{intensity}\;\mathrm{under}\;\mathrm{wound}}{\mathrm{Average}\;\mathrm{collagen}\;\mathrm{intensity}\;\mathrm{of}\;\mathrm{normal}\;\mathrm{dermis}})\;\times \;100$$

### 2.10. Quantitative Histomorphometry

Images for analysis were captured and every section from each specimen was analyzed with MoticBA410 Images Plus2 (Motic, Version 1.1.0.335, Kowloon, Hong Kong, 2013). The number of hair follicles in the skin layer under wound area was determined by counting it through microscopic fields at 40× magnification on three different slides. The fibroblast cells area was also determined by measuring the area that was covered by fibroblast cells instead of inflammatory cells. Both the average number of hair follicles and fibroblast area were expressed in ratio of percentage over to normal dermis as the following Equation (3). On the other hand, the thickness of the epidermis and dermis were determined by measuring the length on the same slide using MoticBA410 Images Plus2 (Motic, Version 1.1.0.335, Kowloon, Hong Kong, 2013)

(3) $$\mathrm{Ratio}\;(\%)\;=\;(\frac{\mathrm{Average}\;X\;\mathrm{under}\;\mathrm{wound}}{\mathrm{Average}\;X\;\mathrm{of}\;\mathrm{normal}\;\mathrm{dermis}})\;\times \;100$$

where *X* represents the number of hair follicles and absolute number of fibroblast cells area.

### 2.11. Assessment of Skin Generation

The wound healing was histologically scored based on the parameters stated in Table 1 [36,37].

### 2.12. Determination of Hematological and Biochemical Endpoints

After eighteen days of treatment, the rats were euthanized, and blood was obtained through cardiac puncture in the right ventricle using a 3 mL syringe. The blood samples were collected as follows: 2 mL of blood was put in a purple ethylene diamine tetra-acetic acid (EDTA) tube for complete blood count and mixed slowly by swirling the tube in order to avoid clotting. The complete blood counts measured were red blood cells (RBC), hemoglobin (Hb), mean red blood cell volume (MCV), mean corpuscular Hb concentration (MCHC), white blood cells, neutrophils, lymphocytes, monocytes, eosinophils, and platelets. Another 2 mL of blood was put into a plain tube for biochemical analysis. The blood samples were allowed to clot for about 30 min at room temperature and centrifuged at 5000 rpm for 5 min to harvest serum (for separation of plasma from blood cells).

The biochemical molecules for routine check-up lists in blood were observed, including protein components (total protein, albumin, globulin), ions (phosphate, calcium), and metabolites (glucose, cholesterol). In addition, organ functions were also examined for the liver (total bilirubin, alanine aminotransferase, alkaline phosphatase), kidney (urea, creatinine), and pancreas (amylase, lipase). Hematology and biochemistry samples were analyzed with automated blood cell counter CELL-DYN 3700 System. Blood samples showing clots or platelets clumping were discarded since the analyte measured could be affected [38].

### 2.13. Statistical Analysis

Data obtained were presented as the mean ± standard deviation (SD), *n =* 3; and analyzed using one-way analysis of variance (ANOVA) and Tukey Pairwise comparisons by Minitab (Minitab Inc., Version 17, State Collage, PA, US, 2018). For one-way ANOVA, only *p*-value < 0.05 was considered as the statistically significant threshold for differences between the groups.

## 3. Results and Discussion

### 3.1. Properties of Palmitoyl-GDPH

The fine powder of Palmitoyl-GDPH was a white color solid mass that is soluble in water. The Palmitoyl-GDPH was further studied for its in vivo wound healing potential since fatty acid conjugated tetrapeptide showed promising results in collagenase and cell migration studies [27]. The toxicity study demonstrated that Palmitoyl-GDPH was not toxic and no mortality was reported over the eighteen days observation period. Additionally, a pH laboratory test was conducted and it was shown that the Palmitoyl-GDPH was acidic when it dissolved in water with pH value of 3.6. We also used circular dichroism to analyze the secondary structure composition and the effect of pH on their confirmation and stability. The stability of Palmitoyl-GDPH was investigated by evaluating the changes in percentage of secondary structure on the difference pH tested (pH 4, pH 7, and pH 10) at 37 °C. Based on the values in Table 2, it was shown that the percentage secondary structures were preferred random structure at all pH levels tested, and this indicates the stability of the peptide.

### 3.2. Effects of Palmitoyl-GDPH on Body Weight

**Figure 2:**
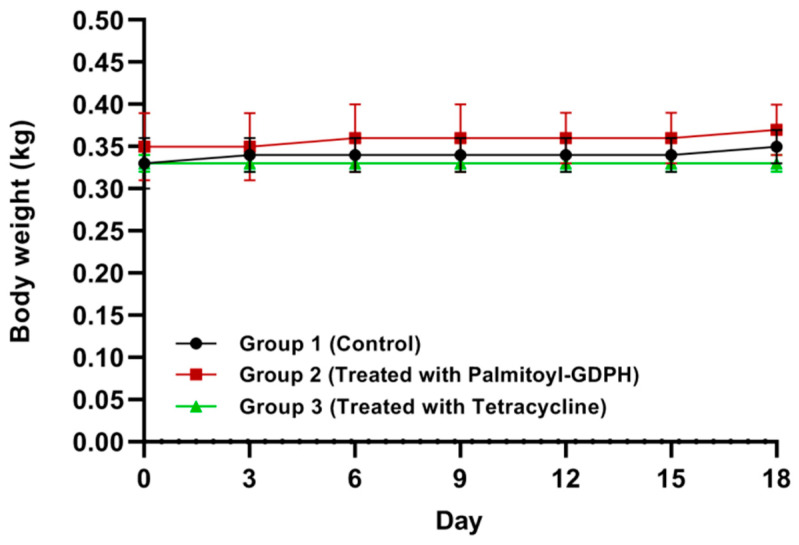
Body weight changes of rats throughout the experimental period.

The observation of physical changes and animal behavior were also important in the in vivo studies. Table 3 shows that there were no abrupt body weight changes in all tested rats. In this study, body weight was monitored as one of the clinical signs. The changes in body weight were plotted against time as shown in Figure 2. Any sudden body weight changes indicated signs of stress or other abnormalities [39]. From the observation, the treated rats were physically active and consumed feed and water in a regular way. There was no adverse reaction to treatment until at the end of experiment. The weight of rats treated with Palmitoyl-GDPH (Group 2) and commercial drug tetracycline (Group 3) were not significantly difference from the control group (Group 1) and remained comparable throughout the experimental time.

### 3.3. Wound Contraction Measurement

**Figure 3:**
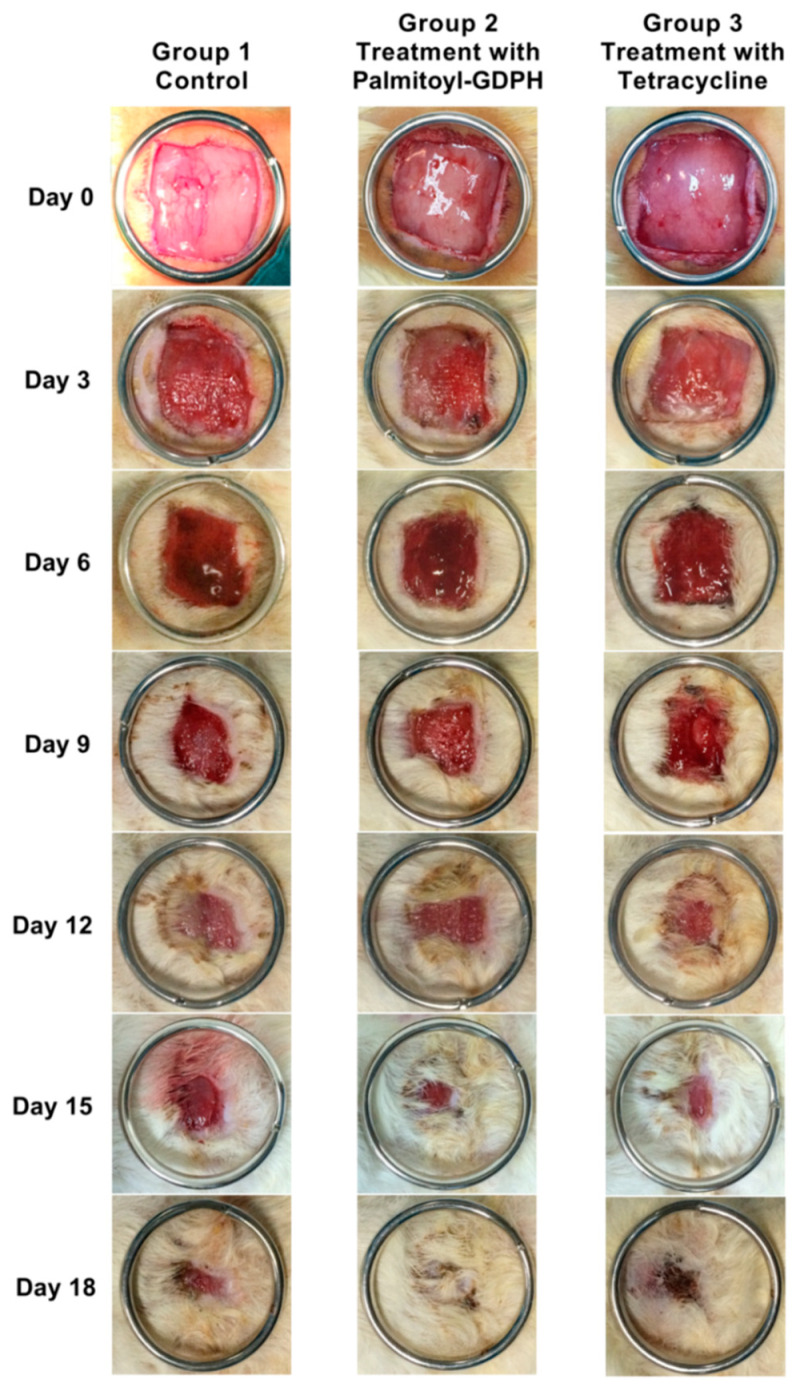
Photograph of representative wound closure profiles for the 18 days experimental period. The 3 cm in diameter ring marks the scale to indicate a reduction in wound size.

**Figure 4:**
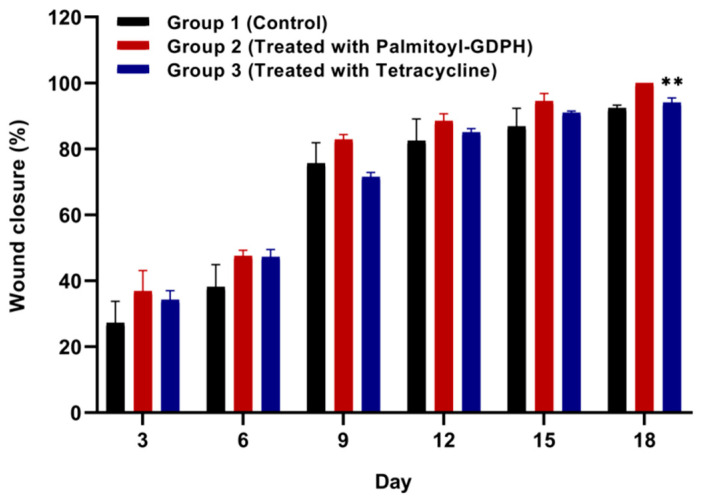
The percentage of wound closure for each group in different days. Data are presented as mean ± SD, *n =* 5. ** Significance at *p* < 0.01 compared with control group.

An in vivo study with a wound model of rats on wound closure was conducted to evaluate the wound healing properties. The progress of wound healing for the skin excision from 4 cm^2^ was followed through the observation of the wound and calculation of the percentage wound reduction. On observation day, the representative image of the wounds and graph of wound area contacted with time from each group are shown in Figure 3 and Figure 4, respectively. The wound area and its rate of reduction varied between the different treated groups over the experimental period. Wound sizes were reduced over a period of time in all groups. The wound treated by Palmitoyl-GDPH (Group 2) shows a higher percentage of wound closure and heals relatively faster in comparison with control and tetracycline groups. On day 15, Palmitoyl-GDPH treated wound shrunk due to re-epithelialization at the wound edge and showed a wound closure of 0.40 ± 0.06 cm^2^ (94.52 ± 2.27%) as compared to control group; with wound area and percentage of wound closure were 0.66 ± 0.20 cm^2^ and 86.85 ± 5.47%, respectively.

Moreover, the wound treated with Palmitoyl-GDPH had a better healing percentage compared to the tetracycline group (Group 3). By day 18, almost 100% of wounds treated by Palmitoyl-GDPH had fully recovered with smooth and flat appearance compared to 94.04 ± 1.44% in the tetracycline group (*p* < 0.01). The control group possessed the minimal wound reduction of 27.27 ± 6.50% on the third day of evaluation and the percentage of wound contraction escalated over the consecutive period of treatment to reach about 92.36 ± 0.91% after 18 days post-surgery. However, the wound bed for the control group was still moist at the central wound region despite the reduction in the wound area over time was observed. This is probably due to the saline that also can promote the healing process by speeding the wound contraction [37,40].

In this in vivo experiment, Palmitoyl-GDPH proved to have the greatest effect on wound treatment compared to vehicle control and being comparable to antibiotic tetracycline likely due to keratinocyte proliferation and migration. Alternatively, the faster healing of the wounds treated with this therapeutic of Palmitoyl-GDPH also could be due to high collagen property thus enhancing cell motility [8]. Based on the previous in vitro study, Palmitoyl-GDPH was shown to be efficient in promoting cell migration and revealed higher collagenase inhibition which may promote wound healing activity. It was suggested that the anti-inflammatory effect of this Palmitoyl-GDPH is the main reason for the enhanced wound healing activity, and this may be related to the increase of connective tissue formation [27]. The topical application has accelerated the wound closure in terms of contraction and re-epithelialization of full-thickness, excisional wound in the rats during the healing process. As a mechanism of action, anti-inflammatory effect of Palmitoyl-GDPH has been suggested to be the main reason for the wound healing activity hence, it could enhance connective tissue formation [27,41,42]. Besides that, since the Palmitoyl-GDPH was acidic when it dissolved in water within the value of pH 3.6, it can further change the wound pH to be relatively more acidic. Even though the previous research showed that Palmitoyl-GDPH does not possess antibacterial properties and does not really inhibit the bacterial colonization [27], we can suggest that this Palmitoyl-GDPH tetrapeptide exerted lethal effect to the bacteria due to its acidic nature promoting healing. By restoring the natural acidic millieu on the skin, it effectively helped in reducing the microbial load on the skin surface. An interventional clinical study also supports this view by showing that topical application of acidic ointments in diabetics significantly reduced their bacterial load on the skin surface [43,44]. Additionally, a similar mechanism was observed with anti-inflammatory effect, cellular proliferation, and migration processes which makes them it a promising therapy for wound healing [45]. Indeed, several studies have tested the effectiveness of therapeutic agents in the treatment of wounds and skin diseases with different formulations and different routes of administration including oral gavage, topical application, and intraperitoneal injection [46,47,48].

### 3.4. Histological Assessment

#### 3.4.1. Hematoxylin and Eosin (H & E) and Masson’s Trichrome Staining

**Figure 5:**
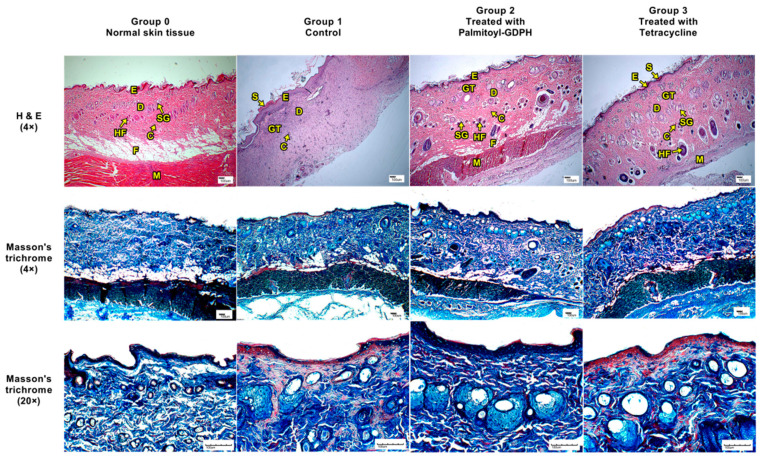
Histological section (H & E) and Masson’s trichrome staining of the healed wound on day 18 post-surgery. The arrow showed epithelialization. S-Scar; E-Epidermis; D-Dermis; GT-Granulation tissue; C-Capillaries; HF-Hair follicle; F-Fat; M-Muscle; SG-Sweat gland. Magnification at 4× (H & E) and 4×, 20× (Masson’s trichrome).

At the end of the experiment, the wound tissues were taken for histological analysis with two types of stain which were H & E and Masson’s trichrome and the evaluations were compared to normal skin. The histological analysis was important for us to observe the detail view of the wound area and to determine the healing effect of different group of treatments. The macroscopic images of H & E and Masson’s trichrome stained tissue sections for normal and test groups are shown in Figure 5.

From the results obtained, the macroscopic appearance of skin from rats control group (Group 1) showed a greater scar, along with the presence of inflammatory cells and minimal sign of collagen deposition since the laid down collagen was scanty with no regular arrangement. However, skin from rats treated with Palmitoyl-GDPH (Group 2) showed no scar, fewer inflammatory cells, more hair follicles, fibroblast and blood vessels, and extensive collagen deposition. Favorably, the Palmitoyl-GDPH treated group demonstrated resemblance to the normal skin tissue (Group 0). Thus, this indicated that Palmitoyl-GDPH treatment had the most complete remodeling outcomes. Comparatively, skin from rats treated with tetracycline (Group 3) showed reduction in scar width, more hair follicles, fibroblast and blood vessels, and moderate collagen deposition.

The underlying mechanism responsible for accelerating wound healing by Palmitoyl-GDPH is due to rapid re-epithelialization which may occurred because of the regulation of collagen expression. Collagen is the most distributed protein in the human body as it is the core component of the extracellular matrix (ECM) which connects the newly generates tissues [49]. The tissue with more collagen deposition suggested a progressive healing of a wound. Moreover, healing activity also depends on angiogenesis process in which the development of new blood vessels. This could improve the crucial transport of oxygen and nutrients which were mandatory for the healing process and re-epithelialization of the wound site. The epithelial cells move across the wound bed and the myofibroblast grips the wound edges thus undergoes contraction to cover the wound area [50,51]. Therefore, these results proved that the Palmitoyl-GDPH treated wound showed good wound contraction and reached complete wound closure on day 18.

**Figure 6:**
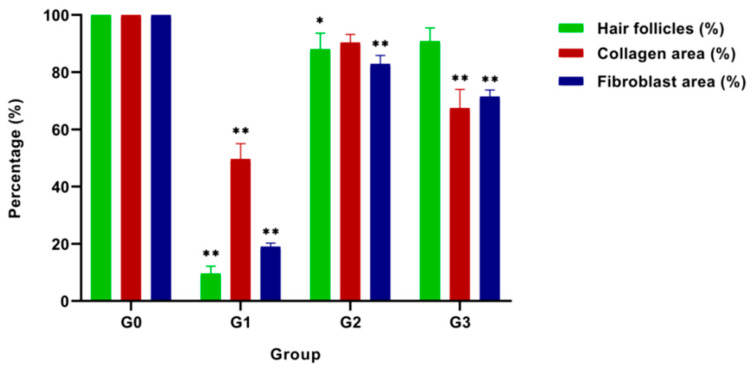
Quantification percentage of hair follicles, area of collagen, and fibroblast cells of healed wound section on day 18 post-surgery. The G0, G1, G2, and G3 represent Group 0 (normal skin tissue), Group 1 (Control), Group 2 (treatment with Palmitoyl-GDPH), and Group 3 (treatment with tetracycline), respectively. Data are displayed as mean ± SD obtained from triplicate experiments. The asterisks (*) represent significant difference (* *p* < 0.05) and very significant difference (** *p* < 0.01) from positive control (normal skin group; Group 0).

Figure 6 represents the quantification of percentage hair follicles, collagen area deposition, and fibroblast area. Accordingly, the Palmitoyl-GDPH group (Group 2) registered the highest scores in the assessment compared to other groups except Group 0. The collagen content in the skin under the wound area treated by Palmitoyl-GDPH was higher, and more fibroblasts cells (82.86 ± 3.00%) and a few hair follicles (88.00 ± 5.61%) were observed. The wound in the control group showed 49.65 ± 5.37% collagen area deposition, whereas the wound treated with Palmitoyl-GDPH and tetracycline showed 90.33 ± 2.91% and 67.47 ± 6.53% collagen deposition, respectively. Morphometric image analysis confirmed the effect of Palmitoyl-GDPH treatment was accelerating the wound healing through the epithelialization of the wound and induced more collagen deposition in the maturation process of wound healing compared to the other treatments. The results indicated that Palmitoyl-GDPH has remarkable wound healing properties.

**Figure 7:**
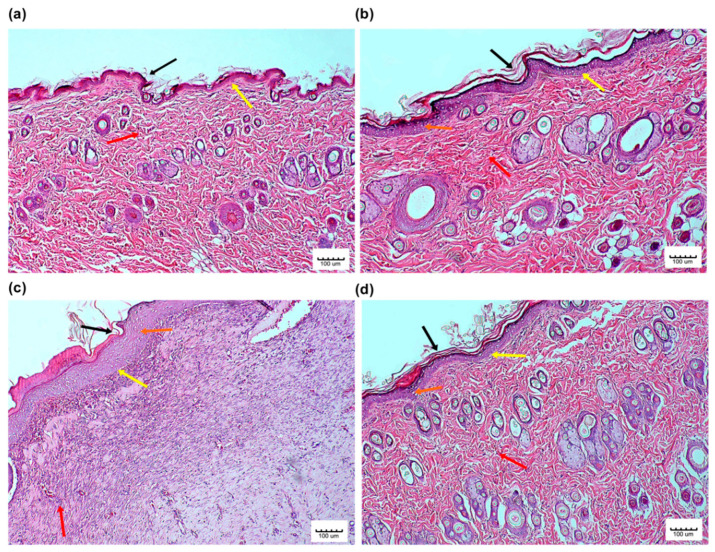
Photomicrograph of skin from the (**a**) normal skin tissue (Group 0); shows an intact tissue integrity especially at the epidermis (black arrow) and epidermal-dermal junction (yellow arrow) along with more fibroblast and collagen deposition (red arrow), (**b**) Palmitoyl-GDPH treated skin tissue (Group 2); shows an almost similar tissue integrity to that of the Group 0 especially the epidermis (black arrow), but a preserved epidermal-dermal junction (yellow arrow), more fibroblast and collagen deposition (red arrow), and normal apoptotic keratinocytes (orange arrow) (**c**) control skin tissue (Group 1); shows the partially destroyed tissue integrity especially the epidermis (black arrow), epidermal-dermal junction (yellow arrow), less fibroblast and plenty collagen deposition (red arrow), and complete apoptotic keratinocytes (orange arrow) and (**d**) tetracycline treated skin tissue (Group 3); shows the well tissue integrity especially the epidermis (black arrow), epidermal-dermal junction (yellow arrow), more fibroblast and collagen deposition (red arrow), and normal apoptotic keratinocytes (orange arrow). Magnification at 10× (H & E).

Moreover, in order to obtain a much more objectively defined results, a quantitative evaluation of selected parameters of wound healing was conducted as previously shown in Section 2.11. The histological image of the normal skin (Group 0) shown in Figure 7a represents a skin that is within normal limits. Our results revealed that the skin tissue especially the epidermal integrity and epidermal-dermal junction in the Palmitoyl-GDPH treated group (Group 2) in Figure 7b presented a better histological appearance compared to control (Group 1 in Figure 7c) and tetracycline (Group 3 in Figure 7d). This could be explained through the ability of the Palmitoyl-GDPH tetrapeptide in promoting wound healing process as well as accelerating the regeneration of epidermis, preserving the structural integrity of skin with replacement by fibroblast, and enhancing collagen deposition.

**Figure 8:**
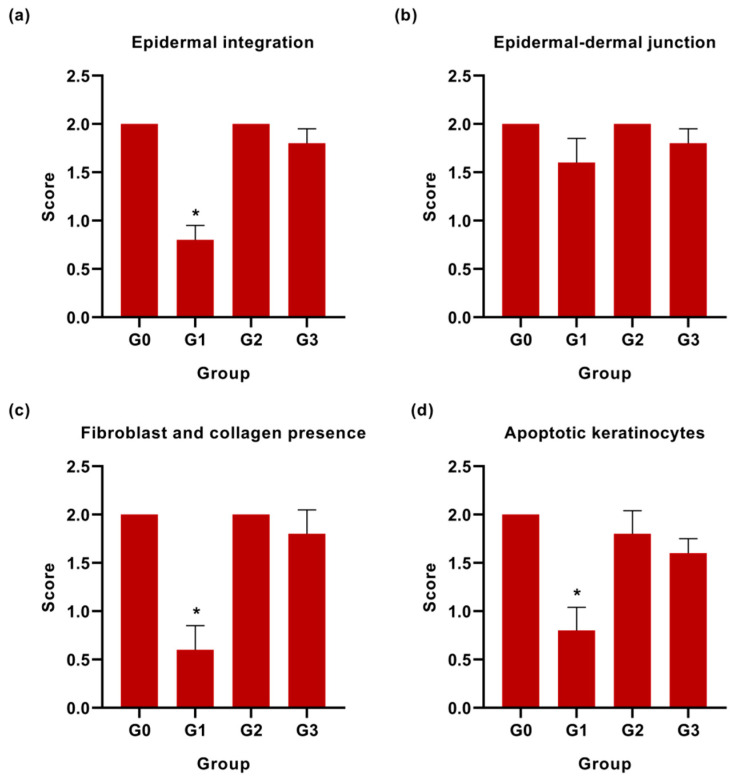
Graphical presentation (mean ± SD) of the histological scoring of (**a**) epidermal integration, (**b**) epidermal-dermal junction, (**c**) fibroblast and collagen presence, and (**d**) apoptotic keratinocytes in skin tissue among four experimental groups. The G0, G1, G2, and G3 represent Group 0 (normal skin tissue), Group 1 (Control), Group 2 (Palmitoyl-GDPH treated skin tissue), and Group 3 (tetracycline treated skin tissue), respectively. The asterisks (*) represent significant difference (* *p* < 0.05) from positive control (normal skin group; Group 0).

At the end of experimental period, no significant difference was seen in the epidermal-dermal junction histology. There was a well-developed epidermal-dermal junction in all groups with an almost clearly defined dermis with many of the intact morphological features. In addition, our results showed a higher apoptotic keratinocyte in the control group (Group 1) as compared to other groups. A previous study reported that inflammatory cells undergo apoptosis, and our findings indicate similar results where the Group 1 also showed a high percentage of inflammatory cells [52]. All the statistical analysis of the microscopically recorded scoring are shown in Figure 8.

#### 3.4.2. Wound Maturity

The cellular content of wound healing component changes is time dependent post-surgery. Therefore, in order to estimate wound maturity, three regions (two marginal regions and one under the wound) of each wound section were analyzed for predominant cell type. Here, wound tissues were obtained at day 18 and its cellular profile was assessed qualitatively in terms the presence of predominant cell type either inflammatory or proliferative.

**Figure 9:**
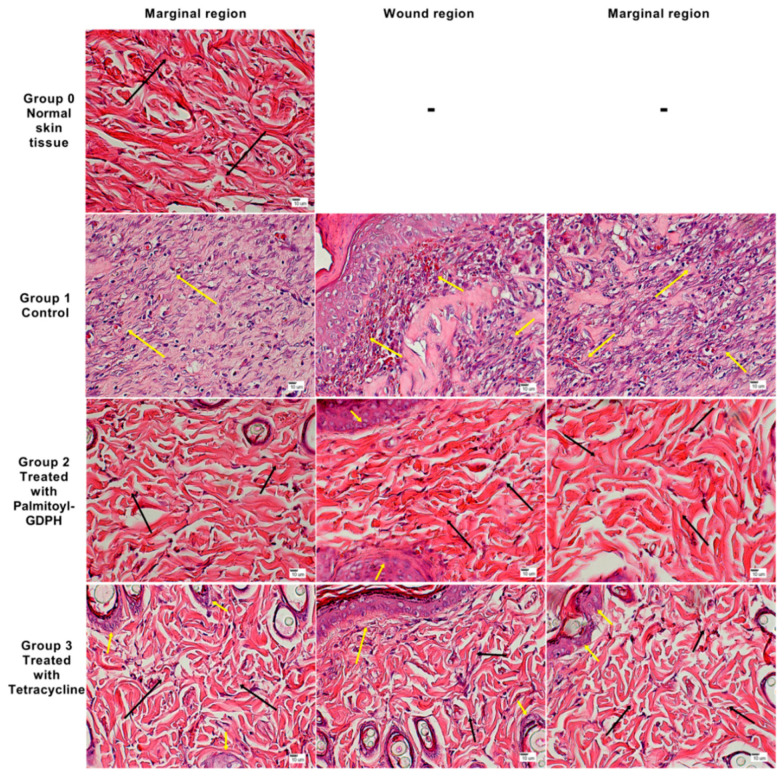
Photomicrograph of H & E stained rat wound section at 40× magnification, showing regions of assessment for predominant cell types that are inflammatory cells (yellow arrow) and proliferative cells (black arrow).

Figure 9 shows the comparison between affected skin layers following wound healing at the end of the experimental period. However, in order to avoid biasness, no images from the normal group (Group 0) was assessed since it was within normal and not subjected to healing (without surgical intervention. The control group (Group 1) showed more purplish discoloration compared to others due to the continuous invasion of inflammatory cells indicating an ongoing healing process, i.e., immature granulation tissue. As opposed to those seen in the Palmitoyl-GDPH group (Group 2) and tetracycline group (Group 3) depicting less intense discoloration, there were fewer fibroblast and proliferative cells. The redness of proliferative cells indicated generation of replacement tissue as the wound healed and matured [53].

#### 3.4.3. Epidermal and Dermal Thickness

**Figure 10:**
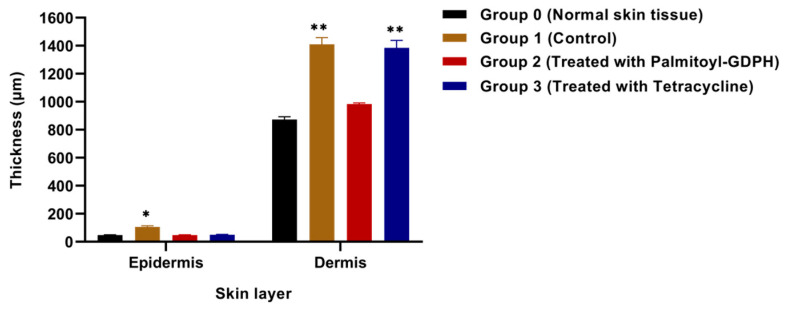
Epidermis and dermis thickness at the end of the experimental period. Data are shown as mean ± SD obtained from triplicate experiments. The asterisks (*) represent significant difference (* *p* < 0.05) and very significant difference (** *p* < 0.01) from normal skin tissue (Group 0).

The alterations in tissue regeneration of the wounded skin were determined by the recovery of the epidermis and dermis of the skin tissue of rats over the designated study duration (18 days). The average thickness of epidermis and dermis skin layer was calculated by measuring the histological morphology of wound area as shown in Figure 10. In the Palmitoyl-GDPH treated group, the epidermis and dermis thickness were 46.99 ± 3.49 µM and 983.52 ± 8.41 µM, respectively, which was near equivalent and almost comparable to the intactness of the normal skin (Group 0). This treatment resulted in much more rapid wound contraction as evidenced by the advanced keratinocytes cell proliferation.

However, the likelihood of the epidermal and dermal thickness of the control group (Group 1) significantly highest (*p* < 0.01) could be due to the absence of re-epithelialization progression from the surrounding wound area towards the center [54]. According to Djemaa et al. (2016), the epidermal thickening observed could presumably be due to the keratinocytes hyper-proliferative activity and their cellular growth [55]. The control wound was completely covered with new epithelium and showed fewer inflammatory cells. The Pamitoyl-GDPH treatment group (Group 2) showed the best healing activity since the layers of epidermis and dermis shows marked regeneration and is well-organized.

### 3.5. Effects of Palmitoyl-GDPH on Hematological Indices

**Figure 11:**
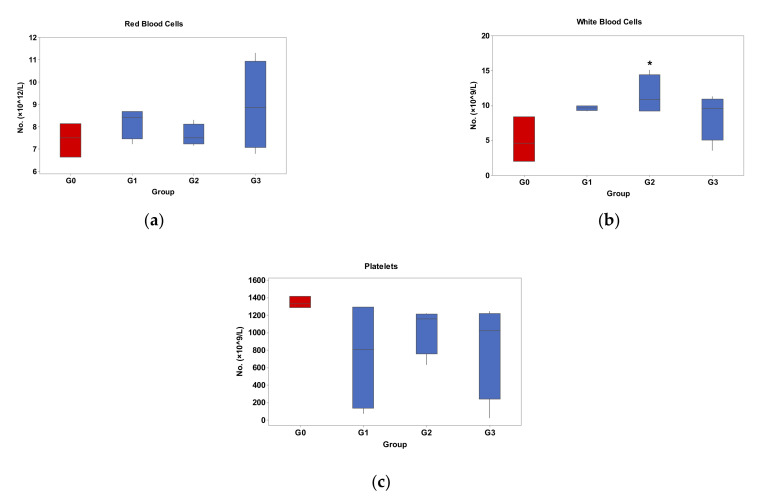
Hematology analysis for 3 major categories of blood cells; (**a**) red blood cells, (**b**) white blood cells and (**c**) platelets. The G0, G1, G2, and G3 represent Group 0 (normal rats), Group 1 (Control rats), Group 2 (Palmitoyl-GDPH treated rats), and Group 3 (tetracycline treated rats), respectively. * Denotes significance (versus normal group) at *p* < 0.05.

The hematology analysis data for individual types of blood cells are presented in Table 4. Moreover, the concentrations of three major categories of blood components, which are red blood cells (RBC), white blood cells (WBC), and platelets were analyzed as in Figure 11. The parameters were paralleled to normal intact rats (Group 0) in order to validly compare toxic changes throughout the experimental period. The results showed that Palmitoyl-GDPH treated group (Group 2) had intermediate numbers of RBC and platelet counts among all the groups. Despite erratic fluctuations in the tetracycline treated group (Group 3), these trends were insignificant compared to the normal group (Group 0) (*p* > 0.05).

However, there was an overall higher (*p* < 0.05) total WBC count in the Palmitoyl-GDPH group (Group 2) than the control, but it remained comparable to other groups. Nevertheless, the true hemogram picture of the Palmitoyl-GDPH group (Group 2) was much lower than the other treated groups when the respective components were compared. Thus, based on the actual WBC (neutrophils, lymphocytes, eosinophils except monocytes) involved, it appears that those in the Palmitoyl-GDPH group (Group 2) have lower counts. Consequently, these lower counts and the slightly higher monocytes are most likely associated with the much faster healing rate whereby WBC counts usually declines [56].

### 3.6. Effects of Palmitoyl-GDPH on Selected Organ Biomarkers

**Figure 12:**
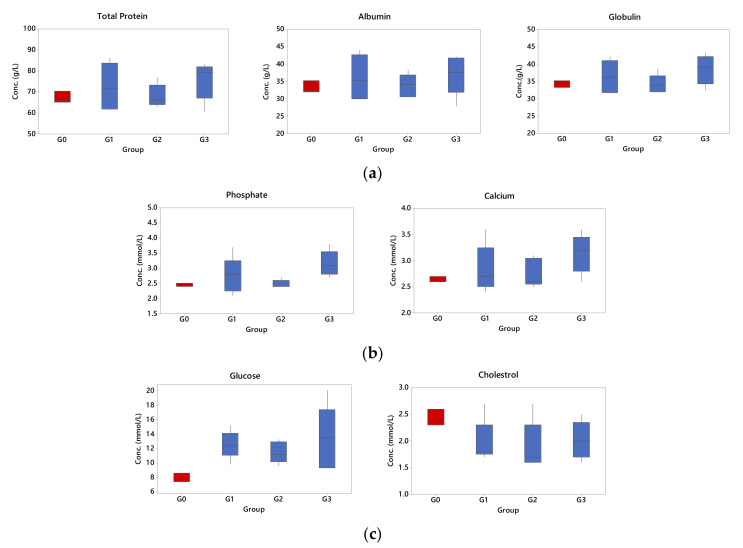
Selected blood chemistry profiles for routine check-up lists in blood; (**a**) Protein components (total protein, albumin, globulin), (**b**) ions (phosphate, calcium), and (**c**) metabolites (glucose, cholesterol). The G0, G1, G2, and G3 represent Group 0 (normal rats), Group 1 (Control rats), Group 2 (Palmitoyl-GDPH treated rats), and Group 3 (tetracycline treated rats), respectively. There is no significant difference from the normal group at *p* > 0.05.

The critical purpose of toxicity analysis was to detect the presence of drug toxicity by various serum biochemical tests. The biochemical molecules in blood were examined using chemical analysis and the data in the parameter details are reported in Table 5. Accordingly, Figure 12 showed chemistry analysis for routine check-up lists in blood after 18 days treatment. The results for protein components (total protein, albumin, and globulin) showed that there was no significant difference between all groups (*p* < 0.05) implying the non-toxic nature of Palmitoyl-GDPH. The concentration of total protein indicates the amount of albumin and globulin in blood. Since proteins were required for development, growth, and health where any derangements maybe exhibited as weight loss [57,58]. As there was no weight loss in all experimental rats further endorses that there was no adverse effect with Palmitoyl-GDPH treatment.

This was also observed for ion components like phosphate and calcium. The results of the treated groups (Groups 1, 2, and 3) were comparable, with no significant difference with the normal group (Group 0) (*p* > 0.05). In contrast, the results of representative metabolites like glucose and cholesterol showed a rather different pattern as depicted in Figure 12c. All the treated groups (Groups 1, 2, and 3) showed a small increase in glucose concentration, while the cholesterol level was lower in the blood compared to the normal group (Group 0). Naturally, the measurement of glucose was very different even for untreated rats and tightly regulated via homeostasis. Consequently, an abnormal level of glucose is the indicator for diabetes and also its concentration can increase due to stress [59,60]. For this case, it is likely elicited of out stress from the wounding and pain. However, our results show the difference in glucose concentration where is still within the reference range and being similar to the normal rats (Group 0).

Biochemical parameters of organ function are sensitive changes in health status and it is important to monitor organ dysfunction. The liver was used as one of the main target organs to detect any toxic effect of Palmitoyl-GDPH applied topically on the skin. Previous works have identified liver involvement in toxicity studies in both rodents and non-rodent species [61,62]. Basic preclinical toxicity testing of liver damage is usually assessed by different blood biochemical parameters. The most common enzymes that are used as signs, indicators or markers of liver function are total bilirubin (TB), alanine aminotransferase (ALT), and alkaline phosphatase (ALP). Damage to the hepatocytes resulted in an elevation in their levels in the blood which is roughly proportional to the degree of tissue injury [63,64].

**Figure 13:**
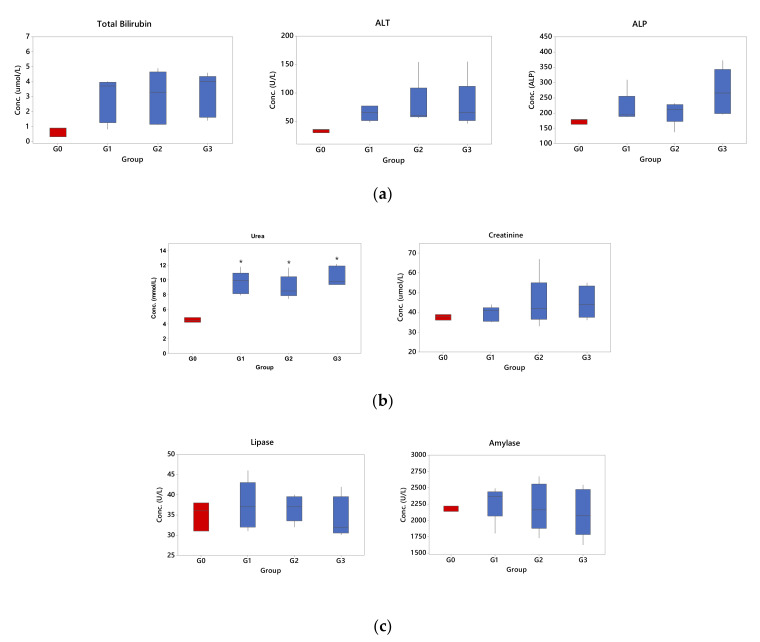
Chemistry analysis for indicators of organ function in blood; (**a**) Indicators for liver function (total bilirubin, ALT, ALP), (**b**) kidney (urea, creatinine) and (**c**) pancreas (amylase, lipase). The G0, G1, G2 and G3 represent Group 0 (normal rats), Group 1 (Control rats), Group 2 (Palmitoyl-GDPH treated rats), and Group 3 (tetracycline treated rats), respectively. The asterisks (*) represent significant difference (* *p* < 0.05) from normal group.

In the act of justifying the safety of topically applied Palmitoyl-GDPH in this study, selected liver, kidney, and pancreatic function biomarkers were determined (Table 6; Figure 13). Based on the results, the values were within reference range limits, although slightly increase in TB, ALT, and ALP values of treated groups (Groups 1, 2, and 3) however, it was insignificantly different from the normal group (Group 0) (*p* > 0.05). Thus, the use of Palmitoyl-GDPH on rats did not show any adverse toxic effects on the liver. In addition, the indicators for kidney (urea and creatinine) and pancreas (amylase and lipase) were analyzed as in Figure 13b,c, respectively. All parameters except for urea were within the stipulated normal range signifying the absence damage inflicted by Palmitoyl-GDPH to the liver and pancreas. Nevertheless, despite registering significant increase in urea concentration in the treated groups (Groups 1, 2, and 3) compared to the normal group (Group 0), the values were still within the normal range. Another reason for an increase in urea not attributed by the tested compounds used may be the different substrate utilization under different physiological conditions [65].

Furthermore, amylase and lipase blood tests were examined to diagnose or monitor pancreatic function. Both involved the enzyme involved in carbohydrate and lipid metabolism, respectively. In the present study, both the results of analysis on the level amylase and lipase in blood revealed comparable concentration level with normal rats (Group 0). No significant differences in amylase and lipase concentration level were observed among all the treated groups (Groups 1, 2, and 3) when compared to the normal group (Group 0).

Overall, data from blood test can be used to infer the general health status of an individual. Thus, if organ damage occurs, there is an increase of the level of components that circulates in the blood. Our findings indicated no significant differences in the specific serum marker levels in rats treated with 12.5 µg/mL of Palmitoyl-GDPH as compared with those of rats in the normal control group. The normal results of blood enzymes confirmed our hypothesis about the non-toxicity of Palmitoyl-GDPH to the organs in vivo. Accordingly, there was no exertion of systemic adverse effects on the animals following wound treatment with Palmitoyl-GDPH.

## 4. Conclusions

Palmitoyl-GDPH revealed remarkable wound healing properties, including accelerated wound contraction and enhanced tissue regeneration. This in vivo wound healing test on rats clearly showed that the topical application of Palmitoyl-GDPH effectively accelerated wound closure. Macroscopic observation further proved that Palmitoyl-GDPH has the ability to heal a wound at a lower concentration compared to standard commercial drug. Histological examination showed that Palmitoyl-GDPH treated group reduced the width of the scar by cell proliferation and collagen formation as well as regenerated skin tissues faster compared to the control group of rats. From hematological and biochemical results, the topical application of Palmitoyl-GDPH on the wound did not result in significant toxicity and no systemic side effect in the liver, kidney, and pancreas of the experimental animals. Overall, Palmitoyl-GDPH is a promising candidate drug as a wound healing therapeutic agent. Additional future works in using this peptide for wound treatment by different physical form such as topical cream and parenteral administration will be beneficial in expanding the current state of knowledge. The safety evaluation of the peptide by in vivo study with examination of the vital organs including lung, heart, liver, kidney, spleen, and brain are required in order to investigate any systemic adverse effect of Palmitoyl-GDPH following the topical application on the wound.

## 5. Patents

The results of the work reported in this manuscript were in patent filed with the title of “A novel fatty acid conjugated tetrapeptide” under Malaysian Intellectual Property Corporation. (PI2018702490).

## Figures and Tables

**Table 1:** Parameters for microscopic assessment of skin generation.

Microscopic Parameter	Score		
	0	1	2
Epidermal integration	Destroyed	Partial	Normal
Epidermal-dermal junction	Destroyed	Partial	Normal
Fibroblast and collagen presence	Plenty	Moderate	A few
Apoptotic keratinocytes	High	Moderate	Normal

**Table 2:** Secondary structure composition of Palmitoyl-GDPH at 37 °C.

Composition	Percentage Secondary Structure Types (%)		
	pH 4	pH 7	pH 10
α-helix	34.40	17.70	0.00
β-sheet	2.60	23.20	34.50
Turn	30.00	20.80	21.10
Random	43.70	38.30	44.40

**Table 3:** Effects of treatment on body weight (*n =* 5).

Group	Rats Taken	Day/Body Weight (kg)						
		0th	3rd	6th	9th	12th	15th	18th
Control (Group 1)	0.28 ± 0.02	0.33 ± 0.03	0.34 ± 0.02	0.34 ± 0.02	0.34 ± 0.02	0.34 ± 0.02	0.34 ± 0.02	0.35 ± 0.02
Palmitoyl-GDPH (Group 2)	0.28 ± 0.02	0.35 ± 0.04	0.35 ± 0.04	0.36 ± 0.04	0.36 ± 0.04	0.36 ± 0.03	0.36 ± 0.03	0.37 ± 0.03
Tetracycline (Group 3)	0.28 ± 0.03	0.33 ± 0.01	0.33 ± 0.01	0.33 ± 0.01	0.33 ± 0.01	0.33 ± 0.01	0.33 ± 0.01	0.33 ± 0.01

**Table 4:** Hematology analysis data for individual types of blood cells. Values are expressed as mean ± SD. The asterisks (*) represent significant difference (* *p* < 0.05) from normal group.

Parameters (Unit)	Group 0	Group 1	Group 2	Group 3
	Normal	Control	Palmitoyl-GDPH	Tetracycline
Red blood cells (×10^12^/L)	7.43 ± 0.75	8.51 ± 0.31	7.39 ± 0.20	8.18 ± 1.56
Haemoglobin (g/L)	150 ± 10.39	154 ± 1.00	157 ± 0.71	153 ± 11.31
Mean red blood cell volume (fL)	52 ± 1.00	52 ± 2.52	54 ± 1.53	49 ± 0.58
Mean corpuscular Hb conc. (g/L)	373 ± 5.66	371 ± 2.00	383 ± 4.93	387 ± 2.52
White blood cells (×10^9^/L)	5.0 ± 3.22	9.8 ± 0.26	10.3 ± 1.73*	10.2 ± 0.99
Neutrophils (×10^9^/L)	0.99 ± 0.90	2.41 ± 0.61	2.33 ± 0.36	3.93 ± 2.16
Lymphocytes (×10^9^/L)	3.65 ± 2.08	6.21 ± 0.55	6.26 ± 0.60	7.86 ± 2.06
Monocytes (×10^9^/L)	0.23 ± 0.17	0.51 ± 0.06	0.72 ± 0.12	0.5 ± 0.33
Eosinophils (×10^9^/L)	0.08 ± 0,08	0.14 ± 0.06	0.24 ± 0.05	0.91 ± 0.95
Platelets (×10^9^/L)	1354 ± 66.34	1295 ± 0.00	1162 ± 1.09	1201 ± 0.78

**Table 5:** Chemistry analysis data for routine check-up lists in blood of protein components. Values are expressed as mean ± SD.

Parameters (Unit)	Group 0	Group 1	Group 2	Group 3
	Normal	Control	Palmitoyl-GDPH	Tetracycline
Total protein (g/L)	67.2 ± 2.78	61.9 ± 0.49	65.9 ± 0.71	76.5 ± 3.89
Albumin(g/L)	33.2 ± 1.77	31.8 ± 2.97	32.8 ± 2.55	38.3 ± 2.86
Globulin (g/L)	34.1 ± 1.03	36.1 ± 3.90	33.5 ± 1.35	38.8 ± 2.52
Phosphate (mmol/L)	2.5 ± 0.06	2.7 ± 0.23	2.4 ± 0.06	2.9 ± 0.20
Calcium (mmol/L)	2.6 ± 0.06	2.6 ± 0.15	2.6 ± 0.06	3.4 ± 0.21
Glucose (mmol/L)	8.1 ± 0.62	12.6 ± 0.36	11.5 ± 0.98	12.5 ± 2.84
Cholestrol (mmol/L)	2.4 ± 0.17	1.8 ± 0.06	1.6± 0.06	1.8 ± 0.2

**Table 6:** Chemistry analysis data for indicators of organ function in blood. Values are expressed as mean ± SD. The asterisks (*) represent significant difference (* *p* < 0.05) from normal group.

Parameters (Unit)	Group 0	Group 1	Group 2	Group 3
	Normal	Control	Palmitoyl-GDPH	Tetracycline
Total bilirubin (µmol/L)	0.7 ± 0.35	3.9 ± 0.15	4.2 ± 0.82	4.2 ± 0.32
ALT (U/L)	34 ± 3.21	73 ± 7.07	61 ± 2.65	63 ± 6.43
ALP (U/L)	173 ± 9.54	193 ± 5.03	222 ± 2.12	317 ± 3.54
Amylase (U/L)	2134 ± 0.71	2360 ± 7.68	2553 ± 5.77	2475 ± 5.90
Urea (mmol/L)	4.5 ± 0.35	10.6 ± 1.04 *	8.7 ± 0.47 *	9.5 ± 0.26 *
Creatinine (µmol/L)	38 ± 1.53	42 ± 1.73	42 ± 1.53	50 ± 5.69
Lipase (U/L)	35 ± 3.61	37 ± 3.51	37 ± 2.00	36 ± 6.03

## Data Availability

Not applicable.
